# Atrial fibrillation catheter ablation complications in obese and diabetic patients: Insights from the US Nationwide Inpatient Sample 2005–2013

**DOI:** 10.1002/clc.23667

**Published:** 2021-06-15

**Authors:** Shawn D'Souza, Mohamed B. Elshazly, Soha R. Dargham, Eoin Donnellan, Nidal Asaad, Sajjad Hayat, Mohamed Kanj, Brian Baranowski, Oussama Wazni, Walid Saliba, Charbel Abi Khalil

**Affiliations:** ^1^ Research Department Weill Cornell Medicine‐Qatar Doha Qatar; ^2^ Joan and Sanford I. Weill Department of Medicine Weill Cornell Medicine New York New York USA; ^3^ Department of Cardiovascular Medicine The Heart Hospital, Hamad Medical Corporation Doha Qatar; ^4^ Biostatistics, Epidemiology, and Biomathematics Research Core Weill Cornell Medicine‐Qatar Doha Qatar; ^5^ Department of Cardiovascular Medicine Heart and Vascular Institute, Cleveland Clinic Cleveland Ohio USA

**Keywords:** AF ablation, diabetes, Nationwide Inpatient Sample (NIS), obesity, postprocedural complications

## Abstract

**Background:**

Obesity and diabetes are risk factors for atrial fibrillation (AF) incidence and recurrence after catheter ablation. However, their impact on post‐ablation complications in real‐world practice is unknown.

**Objectives:**

We examine annual trends in AF ablations and procedural outcomes in obese and diabetic patients in the US and whether obesity and diabetes are independently associated with adverse outcomes.

**Methods:**

Using the Nationwide Inpatient Sample (2005–2013), we identified obese and diabetic patients admitted for AF ablation. Common complications were identified using ICD‐9‐CM codes. The primary outcome included the composite of any in‐hospital complication or death. Annual trends of the primary outcome, length‐of‐stay (LOS) and total‐inflation adjusted hospital charges were examined. Multivariate analyses studied the association of obesity and diabetes with outcomes.

**Results:**

An estimated 106 462 AF ablations were performed in the US from 2005 to 2013. Annual trends revealed a gradual increase in ablations performed in obese and diabetic patients and in complication rates. The overall rate of the primary outcome in obese was 11.7% versus 8.2% in non‐obese and 10.7% in diabetic versus 8.2% in non‐diabetic patients (*p* < .001).

**Conclusions:**

Obesity was independently associated with increased complications (adjusted OR, 95% CI:1.39, 1.20–1.62), longer LOS (1.36, 1.23–1.49), and higher charges (1.16, 1.12–1.19). Diabetes was only associated with longer LOS (1.27, 1.16–1.38). Obesity, but not diabetes, in patients undergoing AF ablation is an independent risk factor for immediate post‐ablation complications and higher costs. Future studies should investigate whether weight loss prior to ablation reduces complications and costs.

## 1 INTRODUCTION

Atrial fibrillation (AF) is the most commonly diagnosed cardiac arrhythmia, predicted to affect 6–12 million people in the US by 2050 and 17.9 million in Europe by 2060.^1^ It is associated with increased risk of death, heart failure, hospitalization, thromboembolic events and an overall impaired quality‐of‐life.^1^, ^2^ Catheter ablation is a potent rhythm control strategy for patients with symptomatic AF^3^ and has been shown to reduce mortality in patients with HF and reduced EF^4^ and improve quality‐of‐life.^5^ Data from clinical trials or academic centers report overall complication rates between 2% and 10% and its safety profile has been improving over the years.^6^, ^7^, ^8^, ^9^

Obesity is a risk factor for the development of AF due to proposed mechanisms such as left atrial enlargement, inflammation and pericardial fat deposition.^10^, ^11^ Diabetes mellitus also is a risk factor, presumably due to increased left ventricular mass and arterial stiffness.^12^ Studies have shown that obesity and diabetes, particularly when poorly managed, are associated with high rates of AF recurrence after ablation.^13^, ^14^, ^15^ Others have shown that risk factor modification reduces the risk of incident and recurrent AF.^16^, ^17^ In terms of procedural complications, meta‐analyses of AF ablation data in obese and diabetic patients primarily from specialized centers have shown comparable rates to non‐obese and non‐diabetic patients.^18^, ^19^ However, the real‐world rate of adverse in‐hospital ablation outcomes in obese and diabetic patients remains largely unknown.

In this study, we aim to use real‐world data from the US Nationwide Inpatient Sample (NIS) to determine annual AF ablation rates in obese and diabetic patients and the independent association of these risk factors with post‐ablation adverse outcomes.

## 2 METHODS

### 2.1 Data source

We used the NIS database to obtain patient data from 2005 to 2013. Since the NIS is an administrative and de‐identified database, it does not require institutional review board approval or exempt determination and informed consent is waived. The NIS is a large publicly available all‐payer inpatient healthcare database designed by the Healthcare Cost and Utilization project to produce US regional and national estimates of inpatient utilization, access, charges, quality, and outcomes accounting for 20% of all US community hospitals. Each entry contains information on demographic details, including age, sex, race, insurance status, primary and secondary procedures, hospitalization outcome, total charges, and length of stay. The NIS contains clinical and resource use information, with safeguards to protect the privacy of patients, physicians, and hospitals.^20^, ^21^ Each admission has its own unique identifiers, so repeat ablations or admissions for the same patient could not be distinguished. With the introduction of the two‐midnight rule by the Centers for Medicare and Medicaid Services (CMS) in 2013, and its full implementation in 2015, most AF ablations have transitioned to observation or outpatient status.^22^ Therefore, we censored our analysis at 2013.

### 2.2 Study population

We used ICD‐9‐CM (International Classification of Diseases, Ninth Revision, Clinical Modification) codes to identify admissions of patients from 2005 to 2013 with a primary diagnosis of AF (code 427.31) who underwent catheter ablation (code 37.34). We excluded patients aged <18, those with a secondary diagnosis of paroxysmal supraventricular tachycardia, paroxysmal ventricular tachycardia, atrial flutter, atrial or ventricular premature complexes, Wolff‐Parkinson‐White syndrome, Long‐Ganong‐Levine syndrome, and atrioventricular nodal tachycardia. We also excluded patients with a history of implantation of a pacemaker or implantable cardioverter‐defibrillator to omit cases of atrioventricular junction catheter ablation for permanent AF. Patients who had open surgical ablation during the hospitalization were also excluded. Similar methods have been used in previous studies analyzing AF ablation in the NIS and other databases.^23^, ^24^

Obese and diabetic (combined uncomplicated diabetes and diabetes with chronic complications) patients were identified using the Agency for Healthcare Research and Quality comorbidity measure for ICD‐9 codes. ICD‐9‐CM codes for body mass index (BMI) were not available for the vast majority of admissions and thus were not used to identify or quantitate obesity given concern for sampling error.

### 2.3 Variables

We used the NIS data elements to identify patient age, sex, race, comorbidities, disposition, hospital volume status, hospital bed‐size, and total admission charges. We defined hospital procedural volume as follows: low volume as <50 AF ablations annually, medium volume as 50–100 AF ablations annually, high volume as >100 AF ablations annually. All hospital charges were inflation adjusted using the price index for the gross domestic product and are reported in 2020 US dollars.

### 2.4 Outcomes

Our primary outcome was the composite of any complication or in‐hospital death. We included vascular complications (post‐op hemorrhage with and without transfusion, blood vessel injury, accidental punctures, AV fistula, retroperitoneal injury, vascular complications needing surgery and others); cardiac complications (iatrogenic cardiac complications, pericardial complications, acute myocardial infarction, requirement for open heart surgery), respiratory complications (pneumothorax, hemothorax, postoperative respiratory failure, and iatrogenic respiratory complications); neurological complications (stroke and transient ischemic attack [TIA]); postoperative infectious complications; and in‐hospital death. Patient safety indicators were used to identify certain respiratory and infectious complications (Table S1). All of these complications were selected based on a review of pertinent clinical literature and identified from corresponding ICD‐9‐CM diagnosis and procedure codes as used by other investigators to maintain consistency,^23^, ^24^ although we added more complications like diaphragmatic injury and pericarditis. Secondary outcomes included in‐hospital mortality alone, length‐of‐stay (LOS), and total inflation‐adjusted hospital charges. We compared these outcomes in obese and non‐obese patients, diabetic and non‐diabetic patients and across four subgroups: non‐obese non‐diabetic, obese non‐diabetic, non‐obese diabetic, and obese diabetic.

### 2.5 Statistical analysis

We used the NIS weights to generate national estimates of the number of admissions each year. We used the χ^2^ test to compare categorical variables for demographics across the four subgroups of non‐obese non‐diabetic, obese non‐diabetic, diabetic non‐obese and obese diabetic, and for comparing the primary outcome between obese versus non‐obese and diabetic versus non‐diabetic patients. We used one‐way ANOVA for age. Generalized linear models were used to analyze annual trends. We also generated nested multivariable models to analyze independent predictors for our outcomes adjusting for different combinations of age, sex, race, hypertension, renal failure, chronic pulmonary disease, peripheral vascular disease, obesity, diabetes, hospital procedure volume, and bed‐size. We used logistic regression used for the primary outcome of any complication including death and secondary outcomes of in‐hospital mortality alone and LOS (0–1 vs. ≥2 days). Linear regression was used for inflation‐adjusted charges, which were initially log transformed. Significance level was set at 5%. All analyses were done using SPSS 26. Cases missing data elements were excluded from analyses.

## 3 RESULTS

After NIS weighing, we identified 106 462 AF ablations performed from 2005 to 2013 who met the inclusion criteria. Table 1 describes the demographics of all patients categorized by obesity and diabetes. The mean age of the overall patient population was 62.8 years (±11.8), 65.2% were male and 89.1% were White. Obese patients accounted for 13 003 (12.2%) and diabetic patients accounted for 17 181 (16.1%) of the population undergoing AF ablation. Compared to patients with neither comorbidity, those with either obesity, diabetes or both had higher rates of chronic diseases and a higher proportion had their ablation performed at low‐volume centers.

**TABLE 1:** Baseline demographics of all AF ablation patients

Demographic variable	All patients *N* (%)	Non‐obese		Obese		*p* values
		Non‐DM *N* (%)	DM *N* (%)	Non‐DM *N* (%)	DM *N* (%)	
All patients	106 462 (100)	80 297 (100)	13 157 (100)	8984 (100)	4019 (100)	
Age ‐ mean (SD)	62.8 (11.8)	62.9 (12.1)	66 (10)	58.8 (10.4)	61.4 (9.3)	<.001
Male	69 368 (65.2)	53 117 (66.3)	8348 (63.4)	5538 (61.6)	2365 (58.8)	<.001
Race						<.001
White	80 053 (89.1)	60 502 (89.8)	9601 (84.7)	6845 (89.7)	3104 (88)	
Black	3013 (3.4)	1855 (2.8)	657 (5.8)	326 (4.3)	175 (5)	
Hispanic	3046 (3.4)	2107 (3.1)	512 (4.5)	294 (3.9)	133 (3.8)	
Asian or Pacific Islander	1152 (1.3)	889 (1.3)	202 (1.8)	25 (0.3)	36 (1)	
Native American	611 (0.7)	495 (0.7)	53 (0.5)	26 (0.3)	37 (1)	
Other	2009 (2.2)	1548 (2.3)	304 (2.7)	113 (1.5)	44 (1.3)	
Hypertension	60 608 (56.9)	40 796 (50.8)	10 055 (76.4)	6525 (72.6)	3231 (80.4)	<.001
Renal failure	5265 (4.9)	2925 (3.6)	1520 (11.6)	313 (3.5)	507 (12.6)	<.001
Chronic pulmonary disease	13 180 (12.4)	8594 (10.7)	2257 (17.1)	1449 (16.1)	880 (21.9)	<.001
Peripheral vascular disease	3781 (3.6)	2409 (3.0)	810 (6.2)	316 (3.5)	246 (6.1)	<.001
Hospital bed size						<.001
Small	4765 (4.5)	3485 (4.4)	663 (5.1)	367 (4.1)	251 (6.3)	
Medium	17 546 (16.6)	13 159 (16.5)	2056 (15.8)	1649 (18.6)	682 (17.2)	
Large	83 328 (78.9)	63 110 (79.1)	10 323 (79.2)	6861 (77.3)	3035 (76.5)	
Hospital volumes						<.001
Low volume (< 50 cases annually)	69 593 (65.4)	50 816 (63.3)	9448 (71.8)	6281 (69.9)	3048 (75.8)	
Medium volume (50–100 cases annually)	19 089 (17.9)	15 140 (18.9)	2043 (15.5)	1377 (15.3)	528 (13.1)	
High volume (>100 cases annually)	17 780 (16.7)	14 341 (17.9)	1670 (12.7)	1325 (14.8)	443 (11)	

Abbreviation: DM, diabetes mellitus.

**FIGURE 1:**
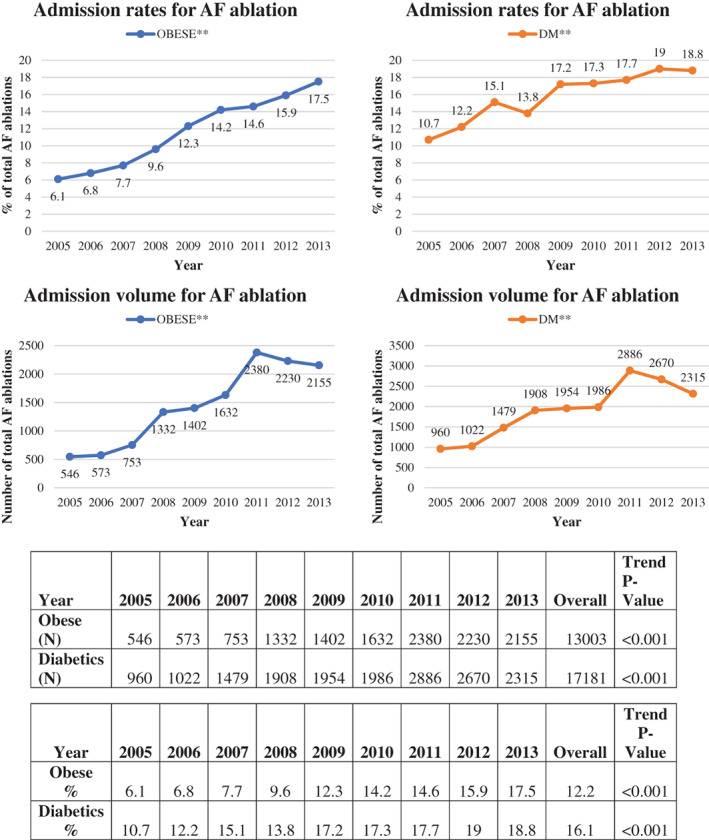
Admission rates and volumes for AF ablation in Obese and Diabetic patients from 2005 to 2013

The annual rates of AF ablations for obese patients gradually increased from 6.1% (*N* = 546) of all AF ablations in 2005 to 17.5% (*N* = 2155) in 2013 (*p* < .001). Similarly, the rates for diabetic patients increased from 10.7% (*N* = 960) of all AF ablations in 2005 to 18.8% (*N* = 2315) in 2013 (*p* < .001). There was a dip in the total number of captured ablations after 2011, likely due to the increased labeling of some of these procedures as outpatient (Figure 1).

**FIGURE 2:**
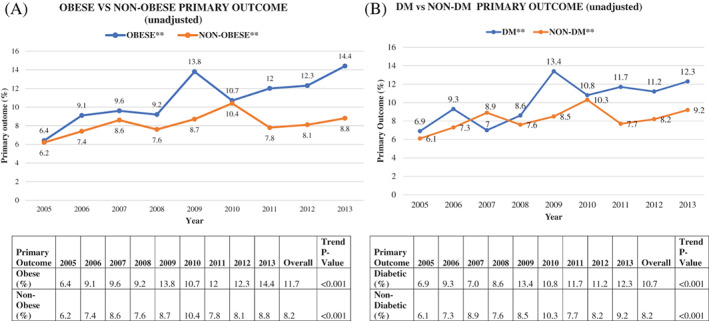
(A) Primary outcome (composite of any complication and death) rates in obese and non‐obese patients from 2005 to 2013. (B) Primary outcome (composite of any complication and death) rates in diabetic (DM) and non‐diabetic (non‐DM) patients from 2005 to 2013. DM, diabetes mellitus

Figure 2(A) shows an increasing rate of the primary outcome (composite of any complication or in‐hospital death) in obese patients from 6.4% in 2005 to 14.4% in 2013 (*p* < .001). Meanwhile, Figure 2(B) shows an increasing complication rate in diabetic patients from 6.9% in 2005 to 12.3% in 2013 (*p* < .001). Tables 2 and 3 describes the annual rates of various grouped complications in obese versus non‐obese (Table 2) and diabetic versus non‐diabetic patients (Table 3). Obese patients showed increasing rates of vascular/hemorrhagic, cardiac, respiratory and infectious complications from 2005 to 2013. Of these, vascular/hemorrhagic, respiratory, and, to a lesser extent, cardiac complications were higher in obese compared to non‐obese patients. Diabetics had higher unadjusted vascular/hemorrhagic, respiratory and neurologic complication rates than non‐diabetics.

**TABLE 2:** Separate complication rates in obese and non‐obese patients

Complication type	Year	2005	2006	2007	2008	2009	2010	2011	2012	2013	Total	Trend *p* value
Vascular/hemorrhagic (%)	Obese	1.8	4.7	4	4.4	6.5	5.5	6.8	5.4	5.8	5.5	<.001
	Non‐obese	3.5	3.5	4.6	4.6	3.9	5.8	3.4	3.6	3.8	4.1	.17
Cardiac (%)	Obese	1.8	1.7	2.1	2.6	4.1	4.3	3.2	2.9	3	3.1	<.001
	Non‐obese	2.1	2.1	3.2	2.6	3.5	3.6	2.9	2.8	3.1	2.9	.001
Respiratory (%)	Obese	0.9	3.3	2.8	2.3	6.1	2.4	4.2	5.8	7	4.5	<.001
	Non‐obese	1.1	1.5	2.2	1.2	1.2	1.8	1.8	1.6	1.7	1.6	.018
Neurologic (%)	Obese	2	0	0.5	0.4	0.7	1.3	1.2	0.2	0.5	0.7	.193
	Non‐obese	0.5	1	0.4	0.6	1	0.8	0.8	0.8	1	0.8	<.001
Infectious (%)	Obese	0	0	0	0.4	0.4	0	0.4	0	0.7	0.3	.016
	Non‐obese	0.1	0.3	0.5	0.2	0.2	0.5	0.2	0.2	0.4	0.3	.217

**TABLE 3:** Separate complication rates in diabetic and non‐diabetic patients

Complication type	Year	2005	2006	2007	2008	2009	2010	2011	2012	2013	Total	Trend *p* value
Vascular/hemorrhagic	DM	3.4	3.5	3.2	3.9	5.9	5.1	4.9	3.9	5.2	4.5	<.001
	Non‐DM	3.4	3.6	4.8	4.7	3.9	5.9	3.7	3.9	3.9	4.2	.011
Cardiac	DM	3.3	2.4	1.2	2.7	3.6	2.7	2.9	2.8	2.6	2.7	<.001
	Non‐DM	1.9	2	3.5	2.6	3.6	3.9	2.9	2.9	3.2	3	<.001
Respiratory	DM	1	2.8	2.8	2.9	3.2	3.4	4.5	4.5	4.8	3.6	<.001
	Non‐DM	1.1	1.4	2.1	1	1.5	1.6	1.6	1.7	2.1	1.6	<.001
Neurologic	DM	1	1.9	0.6	0.3	1.6	1.3	1.9	1.1	0.6	1.2	.005
	Non‐DM	0.6	0.8	0.4	0.6	0.8	0.8	0.6	0.7	1	0.7	.002
Infectious	DM	0	0.4	0.3	0	0.2	1.1	0.3	0	0	0.3	.335
	Non‐DM	0.1	0.3	0.5	0.3	0.2	0.3	0.2	0.2	0.5	0.003	.37

Abbreviation: DM, diabetes mellitus.

There was no significant change in the annual trend in mortality for obese or diabetic patients. The overall mortality rates were 0.1% in non‐obese patients and 0.2% in obese, diabetic, and non‐diabetic groups each. The median LOS in obese patients increased from 1 to 2 days between 2008 and 2009 (*p* < .001). For diabetic patients, the median LOS also increased from 1 to 2 days (*p* < .05). Non‐obese and non‐diabetic patients displayed relatively constant median LOS at 1 day. The total inflation adjusted median hospital charges increased across all groups (*p* < .001). By 2013, obese patients had $9410 higher charges than non‐obese patients. Meanwhile, diabetics showed slightly lower charges than non‐diabetics across all years.

We also sub‐stratified our patient population by obesity and diabetes into four distinct groups. From 2005 to 2013, there has been a relative decrease in AF ablations in patients without obesity or diabetes (84.7% in 2005 to 69.3% in 2013, *p* < .001) (Figure S1) and an increase in annual complication rates across all patient groups (*p* < .001) (Figure S2). However, patients with either obesity, diabetes or both showed much larger increases in the overall complication rate compared to non‐obese non‐diabetic patients. The total inflation adjusted hospital charges for the four groups followed a similar increase across all subgroups. In 2013, obese non‐diabetic patients had the highest median inflation adjusted charges at $105 913 (IQR: $78 277–$138 154), while non‐obese non‐diabetic patients had the lowest at $94 069 (IQR: $66 963–$126 512).

The overall rate of procedural complications or in‐hospital death (primary outcome) (2005–2013) in obese was 11.7% versus 8.2% in non‐obese patients and 10.7% in diabetic versus 8.2% in non‐diabetic patients (*p* < .001). In a multivariate analysis, obesity was found to be an independent predictor of increased rates of the primary outcome (OR [95% CI]: 1.39 [1.20–1.62]), longer LOS (OR [95% CI]: 1.36 [1.23–1.49]) and higher inflation adjusted charges (OR [95% CI]: 1.16[1.12–1.19]) after adjusting for risk factors including diabetes and hospital bed‐size and volume. Whereas, diabetes was associated with longer LOS (OR [95% CI]: 1.27 [1.16–1.38]) but not increased primary outcome or inflation adjusted charges after adjusting for obesity (Table 4).

**TABLE 4:** Multivariate model for primary outcome, complications, in‐hospital mortality, LOS, and inflation‐adjusted charges

	Model 1		Model 2	
Obesity	aOR (95% CI)	*p* value	aOR (95% CI)	*p* value
Primary outcome	1.55 (1.34–1.78)	<.001	1.39 (1.2–1.62)	<.001
Vascular complications	1.35 (1.1–1.64)	.004	1.3 (1.06–1.61)	.013
Cardiac complications	1.1 (0.85–1.42)	.464	1.09 (0.84–1.42)	.524
Respiratory complications	3.52 (2.78–4.46)	<.001	2.66 (2.06–3.42)	<.001
Neurologic complications	1.19 (0.71–2)	.515	1.08 (0.63–1.84)	.781
Infectious complications	2.26 (0.52–2.16)	.716	1.13 (0.49–2.59)	.779
In‐hospital Mortality	0.54 (0.13–2.29)	.405	0.5 (0.12–2.17)	.357
LOS (≥2‐ vs. 0–1‐day)	1.6 (1.46–1.75)	<.001	1.36 (1.23–1.49)	<.001
	Beta (95% CI)	*p* value	Beta (95% CI)	*p* value
Inflation adj. charges	1.16 (1.13–1.2)	<.001	1.16(1.12–1.19)	<.001

DM	aOR (95% CI)	*p* value	aOR (95% CI)	*p* value
Primary outcome	1.28 (1.12–1.46)	<.001	1.1 (0.96–1.26)	.173
Vascular complications	1.07 (0.89–1.29)	.486	0.96 (0.78–1.17)	.66
Cardiac complications	0.88 (0.7–1.13)	.319	0.84 (0.66–1.08)	.18
Respiratory complications	2.26 (1.8–2.86)	<.001	1.59 (1.24–2.03)	<.001
Neurologic complications	1.48 (0.994–2.21)	.054	1.39 (0.92–2.11)	.122
Infectious complications	0.82 (0.4–1.68)	.587	0.6 (0.28–1.28)	.186
In‐hospital Mortality	0.93 (0.39–2.24)	.872	0.84 (0.34–2.1)	.711
LOS (≥2‐ vs. 0–1‐day)	1.55 (1.43–1.68)	<.001	1.27 (1.16–1.38)	<.001
	Beta (95% CI)	*p* value	Beta (95% CI)	*p* value
Inflation adj. charges	1 (0.98–1.03)	.802	0.97(0.95–1.002)	.06

*Note*: Model 1: adjusted for age, sex, and race. Model 2: adjusted for Model 1 + hypertension, renal failure, chronic pulmonary disease, peripheral vascular disease, hospital bed‐size, hospital volume, and obesity or diabetes.

Abbreviation: DM, diabetes mellitus.

In a similar multivariate model assessing individual complication rates (Table 4 **)**, obesity was independently associated with increased rates of vascular/hemorrhagic (OR [95% CI]: 1.38 [1.15–1.66]) and respiratory (OR [95% CI]: 2.66 [2.06–3.42]) complications. Diabetes was associated with increased respiratory complications (OR [95% CI]: 1.59 [1.24–2.03]) only.

Lastly, Figure S3 shows the distribution of AF ablations by hospital volume. AF ablations at low volume centers in our data increased from 46.2% in 2005 to 100% in 2013 (*p* < .001).

## 4 DISCUSSION

In our study analyzing AF ablation admissions in the US from 2005 to 2013 using real‐world data from the NIS, there has been an increasing annual trend of ablations performed on obese (6.1% in 2005 to 17.5% in 2013) and diabetic (10.5% in 2005 to 18.8% in 2013) patients. The overall complication rate, median LOS and inflation adjusted charges have gradually increased, by approximately twofold or more, in both obese and diabetic patients. After adjusting for demographics, clinical variables and hospital size and volume, obesity was associated with 39% increase in complication rates driven mainly by an increase in vascular/hemorrhagic and respiratory complications. Obesity was also independently associated with longer LOS and higher inflation adjusted charges. Diabetes, however, was only associated with increased respiratory complications and longer LOS. Whether aggressive risk factor modification, particularly weight loss, prior to AF ablation reduces ablation costs and complications requires further extensive evaluation in future studies.

Catheter ablation is becoming standard‐of‐care for many AF patients due to its association with improved outcomes and quality‐of‐life^25^ compared to medical therapy. However, it does not come without risks. It is performed while patients are taking oral anticoagulation, and most are still lengthy procedures performed under general anesthesia.^26^ Advanced techniques such as ultrasound guidance for vascular access and trans‐septal puncture have made the procedure safer in patient with comorbidities such as obesity.^27^, ^28^ Nevertheless, these advanced tools are not widely used in low‐volume community centers. NIS real‐world data in our study shows that the number of ablations performed on patients with obesity, diabetes or both has increased over the years, compared to those without both comorbidities, and most were performed in low‐volume centers. Clinical trials and studies from specialized centers have shown that obesity and diabetes were not significantly associated with increased complication rates.^16^, ^17^, ^24^ However, most of these studies involved experienced operators at high‐volume centers. One NIS study from 2000 to 2010 showed an estimated procedural complication rate of 6.29%,^23^ compared to 2.9% reported in a meta‐analysis.^29^ In another study using the national readmission database, 1 in 200 patients died within 30 days and mortality was independently linked to procedural complications.^30^ Although mortality rates in this study were probably inflated due to selection bias prompted by the CMS 2013 two‐midnight rule, it provides insights on the detrimental impact of procedural complications on early mortality. For example, complications due to cardiac perforation or neurological complications were associated with a mortality odds ratio of 2.98 and 8.72, respectively. In our study, we included NIS data only until 2013 to minimize the selection bias resulting from implementation of the two‐midnight rule. We showed increasing annual trends in overall complications in all groups across the 9‐year study period and the magnitude of the increase was much larger in patients with either obesity, diabetes or both compared to those with neither comorbidity. Even if our data is subject to selection bias due to the nature of the NIS, the increasing annual rate of captured admissions for AF ablation in patients with these comorbidities supports our conclusion that they had a higher rate of complication requiring admission. Similarly, an NIS based study from 2017 showed an increase in in‐hospital complication rate for catheter ablation for cardiac arrhythmias from 3.07% in 2000 to 7.04% in 2013 (*p* = .001).^31^ This increase may be attributed partly to changing patient demographics (aging and a greater burden of comorbidities), as well as the increased utilization of complex ablation procedures for AF and VT. In addition, it is likely that an increasing proportion of younger and healthier patients are undergoing catheter ablation procedures on an outpatient basis in 2013, which the NIS does not include as it is an inpatient only database.

In our study, median LOS doubled for most years in patients with either obesity and/or diabetes but remained relatively steady at 1 day for patients without either comorbidity. The total inflation‐adjusted charges associated with inpatient AF ablation gradually increased by twofold across all groups. The largest difference ($ 11 844) in charges was between obese non‐diabetic patients and non‐obese non‐diabetic patients in 2013. This may be due to higher procedural and equipment charges, longer LOS, or higher charges to treat complications. The median total charges associated with inpatient AF ablations in our study are significantly higher than the costs reported in a study of Medicare supplemental databases and MarketScan® commercial claims from 2007 to 2011 (Median cost $25 100 with large facility variation in costs).^32^ These findings may be due to differences in the nature of total charges captured in the NIS, which includes the total amount billed to all payers and not necessarily the actual cost of service or payment received by the hospital. Costs related to catheter ablation include that of the ablation tools (electroanatomic mapping or intracardiac echocardiography‐guided pulmonary vein ablation), hospital and physician billings, and costs related to periprocedural medical care and complications. Reported prices of individual ablation device components (e.g., catheter, ultrasound, needles) reveal a vast range for the cost of a single procedure from $6637 to 12 603 USD (2013) based on the cheapest and most expensive components respectively.^33^ A study from 2014 reports a 22% increase in the cost of AF ablation from 2006 to 2011, far exceeding the rate of healthcare inflation in the US. ^34^ While our study does not delve into the specific reasons for the rising hospital‐billed charges for AF ablation from 2005 to 2013, there is potential for cost reduction through appropriate selection of procedure equipment, standardization of the practice, improved patient selection and further research in new technologies.

We also demonstrate that in all captured AF ablations, obesity was independently associated with increased complication rates (driven primarily by vascular/hemorrhagic and respiratory complications), longer LOS and higher charges after adjusting for demographics, clinical variables, and hospital volume and size. Increased vascular/hemorrhagic complications in obese patients may be due to difficulty in attaining vascular or trans‐septal access even with ultrasound guidance and inadequate hemostasis after catheter removal. Moreover, obese patients undergoing general anesthesia or conscious sedation during this relatively lengthy procedure may experience higher rates of respiratory complications related to worse respiratory mechanics.^35^ Obese patients have been shown to have significantly longer procedural duration than non‐obese patients in a meta‐analysis,^18^ possibly contributing to respiratory complications. The higher costs associated with obesity may be indicative of differences in anesthesia approaches (more general anesthesia rather than sedation) and not just hospital stay and complications. Diabetes, on the other hand, was only associated with worse respiratory complications and longer LOS. Further studies are needed to examine the reasons behind these associations. Other considerations for more studies are differences in operator factors in earlier years such as groin management, anesthesia and anticoagulation strategies that may have affected outcomes, so that further exploration is done to identify the optimal anesthesia and anticoagulation strategies for patients with these risk factors.

The literature extensively describes that poorly controlled obesity and diabetes lead to increased AF recurrence rates after catheter ablation.^13^, ^14^, ^15^ Risk factor modification like weight loss with or without bariatric surgery, glycemic control, blood pressure control, and sleep apnea treatment have been shown to increase arrhythmia‐free survival rates.^36^ We hypothesize that risk factor modification through weight loss prior to AF ablation may also reduce procedural complication rates and charges, but further prospective evaluation is ultimately required. While novel ablation techniques such as pulsed field ablation promise shorter procedures,^37^ we must exercise caution when performing ablations in obese patients, and consistently utilize advanced techniques that guarantee best outcomes such as ultrasound guidance.

Lastly, we want to address the increase of low‐volume hospitals accounting for 100% of AF ablations in the NIS by 2013. During 1988–2011, the NIS was constructed annually by including 100% of the discharges from 20% of US hospitals. However, it was redesigned in 2012 as a 20% national patient‐level sample, with non‐representative sampling across hospitals.^38^ In addition, the introduction of the 2‐midnight rule in 2013 shifted a large proportion of AF ablations to outpatient status.^22^ Since high‐volume centers presumably had fewer complication rates, they likely treated their patients as outpatients or under observation status. Due to a combination of both factors, 100% of AF ablations recorded in the NIS in 2013 seem to be from low volume centers.

## 5 LIMITATIONS

The NIS is a de‐identified administrative database making it difficult to validate individual ICD‐9‐CM codes. This significantly affects the sensitivity and specificity when applying the diagnostic codes. Studies based on data mining are susceptible to errors related to coding. Obesity and diabetes were defined using ICD‐9 codes, which may not be reliable particularly prior to electronic health records. In addition, outcomes related to the severity of obesity were not examined due to insufficient BMI coding in our sample (<10%) and the potential for selection bias. The data from the NIS lacks the level of detail and patient phenotyping available from clinical trials and registries. As previously described, our study is subject to selection bias towards AF ablations requiring admission due to the two‐midnight rule. However, censoring our analysis at 2013 was performed to minimize this bias. This limitation is also offset by the larger sample size from the NIS and the absence of reporting bias introduced by selective publication of results from specialized centers. This unfortunately also means we do not have more recent data from the last 7 years. AF ablation procedures have significantly evolved in the last 7 years leading to improved safety and efficacy, especially in high risk patients, and reduced procedure time as highlighted in contemporary clinical trials such as CABANA, STOP‐AF, CASTLE‐AF, and EAST‐AF4.^4^, ^5^, ^39^, ^40^ Nonetheless, our study highlights the importance of being vigilant about the discrepancies in outcomes reported in clinical trials and those in real‐world clinical practice in the 2005–2013 timeframe, particularly procedures performed at low volume centers. Further studies from contemporary nationwide datasets are needed to evaluate whether these discrepancies in outcomes exist in the current era of AF ablation.

We cannot exclude that confounding variables may have impacted our results. Data about procedural technique, anticoagulation used, medications, and type of AF (i.e., paroxysmal or persistent) were unavailable in the NIS. Late complications like pulmonary vein stenosis and atrio‐esophageal were unavailable for analysis since they do not typically arise during an index hospitalization.

## 6 CONCLUSION

Our study involving real‐world data from the NIS demonstrates increased annual complication rates in patients with obesity or diabetes who underwent inpatient AF ablation from 2005 to 2013. Obesity, but not diabetes, is an independent predictor for higher complication rates, longer LOS and higher hospital charges after adjusting for demographics, clinical variables and hospital volume and size. Granted, our database contains only patients who underwent AF ablation as inpatients from the start or were converted to inpatient after developing complications. Weight loss before ablation may help reduce procedural complications and costs.

## CONFLICT OF INTEREST

The authors declare no potential conflict of interest.

## Supporting information

**Data S1.** Supporting Information.

## Data Availability

This study examined trends in atrial fibrillation catheter ablation outcomes in obese and diabetic patients in the US using discharge data from the Nationwide (National) Inpatient Sample (NIS), Healthcare Cost and Utilization Project (HCUP), Agency for Healthcare Research and Quality (https://www.hcup-us.ahrq.gov/nisoverview.jsp).
