# Supplementary material for: Atrial fibrillation catheter ablation complications in obese and diabetic patients: Insights from the US Nationwide Inpatient Sample 2005–2013
Source: Clin Cardiol. 2021 Jun 15;44(8):1151–60. doi: 10.1002/clc.23667 (PMC8364717; doi:10.1002/clc.23667)
Supplement: Supplementary file 1 — Data S1. Supporting Information. [file CLC-44-1151-s001.docx]

**Atrial Fibrillation Catheter Ablation Complications in Obese and Diabetic Patients: Insights from the US Nationwide Inpatient Sample 2005-2013.**

*Shawn D’Souza, MD,^1^ *Mohamed B. Elshazly, MD,^1,2,3^ Soha R. Dargham, MPH,^1,4^ Eoin Donnellan, MD,^5^ Nidal Asaad, MD,^1,3^ Sajjad Hayat, MD,^1,3^ Mohamed Kanj, MD,^5^ Brian Baranowski, MD,^5^ Oussama Wazni, MD,^5^ Walid Saliba, MD,^5^ Charbel Abi Khalil, MD, PhD^1,2,3^

* First co-authors

Author Affiliations:

^1^ Research department. Weill Cornell Medicine-Qatar. Doha-Qatar

^2^ Joan and Sanford I. Weill Department of Medicine. Weill Cornell Medicine. New York, US

^3^ Department of Cardiovascular Medicine, The Heart Hospital, Hamad Medical Corporation, Doha, Qatar

^4^ Biostatistics, Epidemiology, and Biomathematics Research Core at Weill Cornell Medicine. Doha-Qatar

^5^ Department of Cardiovascular Medicine, Heart and Vascular Institute, Cleveland Clinic, Cleveland, OH, USA

**Corresponding Author:**

Mohamed B. Elshazly, MD, FACC

Weill Cornell Medicine-Qatar.

Office C-125,

Tel: +974-44928324

Fax: +974-44928777

Email: mes2015@qatar-med.cornell.edu

**Supplement Table 1: Inclusion and exclusion criteria, ICD-9 codes**

**Inclusion Criteria:**

Atrial fibrillation (primary position) - 427.31

Ablation of heart tissue via a peripherally inserted catheter (any

position) - 37.34

**Exclusion Criteria :**

Age < 18

Supraventricular tachycardia - 427.0

Ventricular tachycardia - 427.1

Atrial flutter - 427.32

Other premature beats - 427.69

Cardiac dysrhythmia - 427.89

Wolf-Parkinson-White - 426.7

Lown-Ganong-Levine - 426.81

Atrioventricular nodal tachycardia - 426.89

Pacemaker implantation - 00.50, 00.52, 00.53, 37.71 to 37.79, 37.81 to

37.89

Implantable cardioverter defibrillator implantation - 37.94 to 37.98, 00.51, 00.54

Open surgical ablation - 37.33

**Procedural complications:**

**1. In-hospital death**

**2. Vascular/hemorrhagic complications**

- Postop-hemorrhage - 998.11, 998.12. 285.1, 633
- Postop-hemorrhage requiring transfusion - 99.01-99.09
- Vascular complications including:
  - Injury to blood vessels- 900-904
  - Accidental puncture- 998.2, e8700-8709
  - AV fistula-447
  - Injury to retro-peritoneum - 8680.4
  - Vascular complications requiring surgery- 39.31, 39.41, 39.49, 39.52, 39.53, 39.56, 39.57, 39.58, 39.59, 39.79
  - Other vascular complications- 999.2, 997.7

**3. Cardiac complications**

- Iatrogenic cardiac complications - 997.1
- Pericardial complications
  - Hemopericardium – 423.0
  - Cardiac tamponade – 423.3
  - Pericarditis – 420.90
  - Pericardiocentesis – 37.0
- Myocardial infarction – 410.xx
- Requiring open heart surgery - 35.10, 35.11, 35.12, 35.13, 35.14, 35.20, 35.21, 35.22, 35.23, 35.24, 35.25, 35.26, 35.27, 35.28, 35.32, 35.33,35.34, 35.35,35.42, 35.50, 35.51, 35.52, 35.53, 35.54, 35.60, 35.61, 35.62, 35.63, 35.70, 35.71, 35.72, 35.73, 35.81, 35.82, 35.83, 35.84, 35.91, 35.92, 35.93, 35.94, 35.95, 35.96 35.97, 35.98, 35.99, 36.31, 36.32, 37.32, 37.33, 37.35, 37.51, 37.52, 37.53, 37.54

**4. Respiratory complications**

- Pneumothorax/hemothorax - 512.1-512.2, 511.8
- Diaphragm paralysis- 519.4
- Postop-respiratory failure – PSI

| *Acute respiratory failure diagnosis codes for discharges on or after October 1, 2011: (ACURF2D)* | | |
| --- | --- | --- |
| 51851 | Acute Respiratory Failure Following Trauma and Surgery |  |
| *Acute respiratory failure diagnosis codes for discharges prior to October 1, 2011: (ACURFID)* | | |
| 51881 | Acute Respiratory Failure |  |
| *Mechanical ventilation for 96 consecutive hours or more procedure codes: (PR9672P)* | | |
| 9672 | Continuous mechanical ventilation for 96 consecutive hrs or more |  |
| *Mechanical ventilation for less than 96 consecutive hours procedure codes: (PR9670P)* | | |
| 9670 | Continuous Mechanical Ventilation Of Unspec Duration |  |
| *Mechanical ventilation for less than 96 consecutive hours procedure codes: (PR9671P)* | | |
| 9671 | Continuous Mechanical Ventilation For Less Than 96 Consecutive Hrs |  |
| *Reintubation procedure codes: (PR9604P)* | | |
| 9604 | Insertion Of Endotracheal Tube |  |

- Other iatrogenic respiratory complications - 997.3

**5. Neurological Complications :**

- Postop-Stroke/TIA - 997.0, 997.00, 997.01, 997.02, 435.9, 438.0, 438.10, 438.11, 438.12, 438.19, 438.20, 438.21, 438.22, 438.30, 438.31, 438.32, 438.40, 438.41, 438.42, 438.50, 438.51, 438.52, 438.53, 438.81, 438.82, 438.89, 438.9

**6. Infection:**

- Post-procedural aspiration pneumonia - 997.32
- Postop infectious complications – PSI

| For discharges on or after October 1, 2007, but before October 1, 2011, the selected infection is: | |
| --- | --- |
| *Central venous catheter-related blood stream infection diagnosis codes: (IDTMC2D)* | |
| 99931 | Infection due to central venous catheter |
| For discharges on or after October 1, 2011, the selected infection is: | |
| *Central venous catheter-related blood stream infection diagnosis codes: (IDTMC3D_PSI)* | |
| 99932 | Bloodstream infection due to central venous catheter |

*Sepsis diagnosis codes for discharges in FY2007 and later: (SEPTI2D_PSI)*

| 0380 | Streptococcal septicemia | 03843 | Pseudomonas |
| --- | --- | --- | --- |
| 0381 | Staphylococcal septicemia | 03844 | Serratia |
| 03810 | Staphylococcal septicemia, unspecified | 03849 | Septicemia due to other gram-negative  organisms |
| 03811 | Meth susc staph aur sept | 0388 | Other specified septicemias |
| 03812 | Mrsa septicemia | 0389 | Unspecified septicemia |
| 03819 | Other staphylococcal septicemia | 78552 | Septic shock |
| 0382 | Pneumococcal septicemia (streptococcus  pneumoniae septicemia) | 99591 | Systemic inflammatory response  syndrome due to infectious; |
| 0383 | Septicemia due to anaerobes | 99592 | Systemic inflammatory response  syndrome due to infectious; |
| 03840 | Gram-negative organism, unspecified | 9980 | Postoperative shock /* not valid after  october 1, 2011 |
| 03841 | Hemophilus influenzae | 99802 | Shock following trauma or surgery, septic |
| 03842 | Escherichia coli |  |  |

**For all figures, ** = p<0.001, *= P<0.05**

**Supplement figure 1: AF ablation rates from 2005-2013 sub-stratified by obesity and diabetes**

| **Admission rate (%)** | **2005** | **2006** | **2007** | **2008** | **2009** | **2010** | **2011** | **2012** | **2013** | **Overall** | **Trend**  **P-Value** |
| --- | --- | --- | --- | --- | --- | --- | --- | --- | --- | --- | --- |
| **Non-Obese non-DM** | 84.7 | 82.4 | 79.8 | 79 | 74.1 | 73.3 | 72.3 | 70.5 | 69.3 | 75.4 | 0.098 |
| **Obese non-DM** | 4.5 | 5.4 | 5.1 | 7.2 | 8.7 | 9.4 | 10 | 10.4 | 11.9 | 8.4 | <0.001 |
| **DM non-Obese** | 9.2 | 10.8 | 12.5 | 11.3 | 13.6 | 12.5 | 13.1 | 13.6 | 13.2 | 12.4 | <0.001 |
| **Obese DM** | 1.6 | 1.4 | 2.5 | 2.5 | 3.6 | 4.8 | 4.6 | 5.5 | 5.6 | 3.8 | <0.001 |

**Supplement figure 2: Primary outcome rates from 2005-2013 sub-stratified by obesity and diabetes**

| **Primary  Outcome (%)** | **2005** | **2006** | **2007** | **2008** | **2009** | **2010** | **2011** | **2012** | **2013** | **Overall** | **Trend**  **P-Value** |
| --- | --- | --- | --- | --- | --- | --- | --- | --- | --- | --- | --- |
| **Non-Obese non-DM** | 6.1 | 7.2 | 8.8 | 7.5 | 8.1 | 10.2 | 7.2 | 7.6 | 8.3 | 7.9 | 0.007 |
| **Obese non-DM** | 7.4 | 8.3 | 10.3 | 8.8 | 12.3 | 11.4 | 11.2 | 11.9 | 14.7 | 11.4 | <0.001 |
| **DM non-Obese** | 7.4 | 9 | 6.8 | 8.3 | 12.3 | 11.4 | 11 | 10.5 | 11.7 | 10.1 | <0.001 |
| **Obese DM** | 3.6 | 11.9 | 8.1 | 10.2 | 17.5 | 9.1 | 13.7 | 13.1 | 13.8 | 12.3 | <0.001 |

**Supplement figure 3: Admission rates for AF ablation from 2005-2013 sub-stratified by hospital volume**
